# Overdiagnosis of Bipolar Disorder: A Critical Analysis of the Literature

**DOI:** 10.1155/2013/297087

**Published:** 2013-11-20

**Authors:** Amna A. Ghouse, Marsal Sanches, Giovana Zunta-Soares, Alan C. Swann, Jair C. Soares

**Affiliations:** UT Center of Excellence on Mood Disorders, Department of Psychiatry and Behavioral Sciences, The University of Texas Health Science Center at Houston, UT Houston Medical School, 1941 East Road, Houston, TX 77054, USA

## Abstract

Bipolar disorder (BD) is considered one of the most disabling mental conditions, with high rates of morbidity, disability, and premature death from suicide. Although BD is often misdiagnosed as major depressive disorder, some attention has recently been drawn to the possibility that BD could be overdiagnosed in some settings. The present paper focuses on a critical analysis of the overdiagnosis issue among bipolar patients. It includes a review of the available literature findings, followed by some recommendations aiming at optimizing the diagnosis of BD and increasing its reliability.

## 1. Introduction

Bipolar disorder (BD) is a chronic, recurrent mental illness first described by Jules Falret in 1854 as Folie Circulaire (circular insanity). Later, it was renamed as manic-depressive Psychosis. Nowadays, BD is considered one of the most disabling mental conditions, with high rates of morbidity, disability, and premature death from suicide [1]. Although the traditionally accepted prevalence of BD ranges from 0.5 to 1.5%, more recent epidemiological studies suggest that these rates may be as high as 10%, depending on which diagnostic criteria are adopted. 

These discrepancies in prevalence point to a lack of consensus on the current definitions of BD, which may favor the overdiagnosis of major depressive disorder (MDD) in detriment of BD [2]. Based on the literature findings, the rates of bipolar patients mistakenly diagnosed with MDD ranges from 10 to 40% [3, 4], with some authors suggesting even higher proportions of misdiagnosis. For example, one study reported that almost 60% of bipolar patients were initially misdiagnosed with other conditions, particularly MDD [5].

Over the last decade, however, BD has been progressively incorporated by culture. Patients with BD are now commonly displayed in the media, and allusions to this condition are frequently found not only in the scientific literature but also in autobiographical books and fictional writing. This trend has drawn attention to the possibility that, regardless of well-demonstrated rates of underdiagnosis, BD could be overdiagnosed in some settings. 

 The potential downsides of overdiagnosing BD are several. They include the negative effects of unnecessary labeling, the risk of harm related to unnecessary treatments, and the misuse of health care resources, with important human and financial implications [6–8].

 The present paper comprises a critical analysis of the overdiagnosis issue among bipolar patients. We begin with a review of the available literature findings, followed by some recommendations aiming at optimizing the diagnosis of BD and increasing its reliability.

## 2. Methods

The database PubMed (1990–2013) was searched, using the MeSH terms “bipolar disorder” and “diagnosis.” In addition, we carried out a manual search of bibliographical cross-referencing. The papers obtained were manually screened, aiming at identifying original articles, reviews, and case reports focusing on the overdiagnosis of BD. 

## 3. Rates of Overdiagnosis of BD: A Review of the Evidence

 We identified seven studies that included data on the overdiagnosis of BD (Table 1). The results of these studies point to considerable variability in the rates of overdiagnosis, which seems to range from 4.8 to 67%. Most of the studies identified focused on outpatient populations and addressed the misdiagnosis of BD among patients with other psychiatric conditions (e.g., MDD and attention-deficit disorder). 

Two of those studies [9, 13] utilized the structured clinical interview for DSM-IV disorders (SCID) as a gold standard and included that on the positive and negative predictive value of the diagnosis of BD made by clinicians. The evidence suggests that while clinician's diagnosis has a high negative predictive value, its positive predictive value is relatively low. In other words, if a psychiatrist had not diagnosed a patient with BD, most likely the patient did not have that diagnosis as per the SCID, whereas if a clinician diagnosed a patient with BD, the chance that the patient effectively met criteria for that diagnosis according to the SCID was modest. The implications of those findings are addressed in Section 5 of the present paper.

## 4. Factors Involved in the Overdiagnosis of Bipolar Disorder

### 4.1. Personality Disorders as a Possible Confounder in the Diagnosis of Bipolar Disorder

 The phenomenological distinction between BD and some personality disorders may be challenging, not only because of the overlap between some personality disorder features and the diagnostic criteria for mood episodes but also because of the lack of reliability of the time criteria for BD. For example grandiosity is a common feature in narcissistic personality disorder, and patients with histrionic personality traits can be mistakenly diagnosed with mania [12]. Anger and impulsivity, often present in patients with antisocial personality disorder, may be misinterpreted as irritable mood [15]. 

 Maybe the most important debate regarding the interface between BD and personality disorders is related to the issue of differential diagnosis versus comorbidity. That is particularly true when it involves patients with borderline personality disorder (BPD). While the cooccurrence of BPD and BD seems to be quite common [16], the similarity between borderline personality and bipolar disorder may contribute to the diagnostic confusion involving these two conditions. Instability is a core feature in BPD, and one of the most common complaints among these patients is the occurrence of “mood swings.” Although there are major differences between the affective instability of BPD patients and the classic cycling patterns of BD, some authors pointed to the existence of “ultrarapid cycling” among BD patients, which would be virtually indistinguishable from the mood shifts reported by patients with BPD. Moreover, impulsivity, a hallmark of BPD, is also increased among bipolar patients, even during periods of euthymia [17, 18]. In a recent study, nearly 40% of BPD patients were found to have a mistaken diagnosis of BD [8], whereas other studies reported an even higher rate (56%) of over diagnosis of BD [12].

 On the other hand, the multiple similarities between the two disorders have raised the issue that BD and BPD actually belong to the same spectrum, therefore representing different presentations of the same basic condition [19]. While this point of view is not supported by the current psychiatric nosology, it can affect clinician's diagnostic rationale, contributing to the blurring of the diagnostic limits between these two conditions [20].

### 4.2. Substance Use Disorders and the Diagnosis of Bipolar Disorder

 The rates of comorbidity between substance use disorders and BD are extremely high [21]. On the other hand, the behavioral effects of several psychoactive substances can easily mimic the mood symptoms usually found in patients with bipolar disorder. Patients often describe typical manic or hypomanic symptoms associated with the use of cocaine and stimulants, and alcohol has well-described depressive effects on mood. Although DSM-IV recommends the diagnosis of mood disorder secondary to substances in patients whose mood symptoms seem to be restricted to periods of substance use, this distinction can be difficult in clinical practice. In a study assessing 21 patients diagnosed by their clinicians as suffering from BD and comorbid substance abuse or dependence, it was demonstrated that only 9 (42.9%) actually met full criteria for BD as per a structured diagnostic interview [10]. Similar findings were obtained by another study [22] which retrospectively analyzed the records of patients with BD admitted to a dual diagnosis inpatient unit. The authors found that only 33% of the patients with a previous diagnosis of BD effectively met criteria for that condition.

### 4.3. Attention-Deficit Hyperactivity Disorder and BD in Children

 There is a marked trend toward increases in the diagnosis of mental disorders in children [23, 24]. Specifically in regard to BD, a recent study documented a fortyfold increase in the diagnosis of bipolar disorder in children [25]. ADHD symptoms, such as irritability, rapid or impulsive speech, physical restlessness, impaired attention, and sometimes defiant or oppositional behavior, can be mistakenly interpreted as resulting from BD [26, 27]. It has been suggested that some symptoms such as grandiosity, elated mood, hypersexuality, flight of ideas, and decreased need for sleep can help in the differentiation between BD and ADHD [28]. BD should also be suspected in the presence of psychotic symptoms, severe mood swings, sudden-onset or late-onset (after age 10) ADHD symptoms, strong family history of BD, and lack of response to stimulants in a child with a history of positive response to them [29].

### 4.4. Limitations of the DSM-IV Criteria for Bipolar Disorder

DSM-IV criteria have been widely used for the diagnosis of psychiatric conditions over the last two decades. Although the reliability of its criteria for the diagnosis of BD, especially in the presence of mania, has been extensively demonstrated, there is evidence suggesting that some of its criteria may lack validity [30]. A recent study using combinatorial analysis [31] highlighted that, given the extremely high number of possible criteria combinations in order to characterize core mood episodes, the validity of the DSM-IV diagnosis of BD may be questioned.

Furthermore, some of the criteria used to characterize manic or hypomanic episodes are unspecific, such as decreased concentration, racing thoughts, and psychomotor agitation. These symptoms can be found in several different conditions, such as anxiety disorders and attention-deficit disorder. Moreover, the distinction between hypomania and nonpathological behavior is often problematic, which can sometimes result in the overdiagnosis of BD type II. 

 Perhaps the most important limitation of the DSM-based diagnosis of BD comes from its inability to identify bipolar patients without a typical history of classic manic or hypomanic symptoms, that is, a patient whose index episode is depressive. Attempts have been made to minimize this limitation through the description of features that, if present in a patient with depression, are highly suggestive of bipolar disorder [32]. Among these features, we can find family history of BD, psychotic symptoms, atypical features, history of manic/hypomanic symptoms induced by antidepressants, and postpartum onset [33]. In its original form, this concept helps alert clinicians in regard to a possible diagnosis of BD, reducing the risk of underdiagnosis. On the other hand, it is possible that the misinterpretation of the appropriate relevance of these features may contribute to the misclassification of unipolar patients as suffering from BD in certain circumstances [32].

### 4.5. The “Bipolar Spectrum” Concept

 The concept of “bipolar spectrum” represents the antithesis of the issues exposed above in regard to the limitations of DSM-IV criteria for BD. Based on a dimensional approach, it expanded the diagnosis of BD beyond the categorical framework established by DSM-IV, based on the assumption that individuals with a strong genetic load for BD can experience different varieties of bipolarity. Consequently, many patients who do not meet criteria for BD based on the DSM-IV criteria may be diagnosed as suffering from “soft” forms of BD, which sometimes can overlap with the patient's temperament [34]. Of notice, as the concept of bipolar spectrum is progressively expanded, the diagnosis of unipolar depression becomes extremely restricted and tends to disappear. 

 Despite the controversy surrounding this concept, it has gained immense popularity over the last decades, and many clinicians adopt this model to formulate (or sometimes justify) their diagnosis of BD. Despite sometimes minimizing the diagnostic labeling represented by a categorical approach, a dimensional diagnostic model (such as the one represented by the bipolar spectrum concept) tends to blur the boundaries between different diagnostic concepts and normalities, decreasing even more the diagnostic reliability across different providers [35].

### 4.6. Other Issues

There are several additional possible reasons for overdiagnosis. For example, disability awards especially due to mental illness are on the rise. Between 1987 and 2005, the share of Supplemental Security Income's adult caseload disabled due to a mental disorder rose from 24.1% to 35.9%. In a recent study, 172 patients on disability due to a diagnosis of BD were assessed, and only 47.6% of them actually met criteria for BD [13]. 

## 5. Optimizing the Diagnosis of BD

 The recent publication of DSM-5 was expected by many as a promising step in improving the diagnostic accuracy of BD. Increase in energy and activity was included as a core symptom for the diagnosis of mania or hypomania, in addition to elated mood. Mixed episodes were removed from DSM-5. Instead, the presence of mixed features became a specifier, which may be present among patients with manic or depressive episodes [36]. It is still early to assess the impact of these changes on the diagnosis of BD by clinicians. 

 On the other hand, the medical literature is scant in regard to interventions aiming at minimizing the overdiagnosis of BD. In a recent paper [37] it was argued that the low specificity of the diagnosis of BD based on structured instruments/criteria (including the DSM proposed criteria for BD) is inherent to the relatively low prevalence of the disorder in the population in question, which automatically implicates in a lower predictive positive value of the criteria in question. The authors conclude that the goal of improving the diagnostic accuracy of BD can be accomplished not by improving the specificity or increasing the diagnostic threshold of that condition, but through increasing the prior probability of the diagnosis before applying diagnostic criteria. This could be accomplished, for example, by educating clinicians in regard to the identification of soft signs of bipolar disorder, careful documentation of family history of bipolar disorder, and close attention to features suggestive of underlying bipolarity among patients with depression.

 The “two-step diagnostic process” described above could also be accomplished through the systematic administration of a screening instrument for BD, such as the Mood Disorder Questionnaire [38] prior to applying structured criteria. Screening instruments usually display high sensitivity but low specificity and can therefore aggravate the problem of overdiagnosis if isolatedly applied, since clinicians tend to interpret a positive screening as indicator of diagnosis, again ignoring the fact that the positive predictive value of a screening tool is directly correlated with its prevalence. 

 Furthermore, over the last decades, considerable advances have been made in the better understanding of the pathophysiology of BD through neuroimaging studies, analysis of neuropsychological findings among bipolar patients, genetic analysis, and several other biological measures. Whereas it is expected that these progresses bring about increments in the diagnosis of BD in the long term, it is unlikely that a single biomarker might be able to identify patients with BD, given the biological and clinical heterogeneity of that condition [39]. On the other hand, considerable research has recently focused on an integrative approach, aiming at the identification of biosignatures, which would encompass, for example, findings regarding neurocircuitry abnormalities, measures of oxidative stress, and genetic markers [39]. Despite how promising this approach might sound, it is still in its childhood [40].

## 6. Conclusions

 Given the current status of BD as a construct, diagnostic errors and the mistaken identification of patients as suffering from that condition are likely to occur. Nevertheless, improvements in the nosological and psychopathological characterization of BD, as well as clinician education on the correct interpretation of the different factors involved in the detection of BD, may increase the reliability of that diagnosis. Similarly, the identification of biological markers for mood disorders represents a promising area of research and will likely bring about improvements in the precision of the BD diagnosis in the near future.

## Figures and Tables

**Table 1:** Studies addressing the overdiagnosis of bipolar disorder.

Author/year	Sample	Gold standard	Findings
Ghaemi et al. (2000) [9]	Outpatients	SCID	Clinician-based diagnosis of BD: PPV of 34% and NPV of 95%
Stewart and El-Mallakh (2007) [10]	Outpatients from a substance abuse treatment program	DSM-IV criteria	Only 42.9% of patients diagnosed with BD actually met diagnostic criteria
Goldberg et al. (2008) [11]	Dual diagnosis inpatients	SCID	Only 33% of patients diagnosed with BD actually met criteria for that condition. Misdiagnosis associated with cocaine and polysubstance abuse
Zimmerman et al. (2008) [8]	Outpatients	SCID	Clinician-based diagnosis of BD: PPV of 37% and NPV of 95%
Ruggero et al. (2010) [12]	Outpatients	SCID	40% of patients with borderline personality disorder mistakenly diagnosed with BD
Zimmerman et al. (2010) [13]	Outpatients	SCID	Patients overdiagnosed with BD were significantly more likely to receive disability payments
Chilakamarri et al. (2011) [14]	Child outpatients	DSM-IV criteria	Minimum number of patients misdiagnosed with BD; underdiagnosis was common

BD: bipolar disorder, SCID: structured clinical interview for DSM-IV disorders; PPV: positive predictive value; NPV: negative predictive value.
